# Professional Quality of Life among healthcare workers in a Covid-19 unit

**DOI:** 10.1192/j.eurpsy.2023.935

**Published:** 2023-07-19

**Authors:** I. Sellami, A. Haddar, A. Abbes, H. Halweni, M. L. Masmoudi, K. Jmal Hammami, M. Hajjaji

**Affiliations:** 1 occupational medecine, Hedi Chaker hospital, University of Sfax; 2 Family department, university of Sfax, Sfax, Tunisia

## Abstract

**Introduction:**

The pandemic of Covid-19 has overwhelmed healthcare systems including healthcare workers(HCWs) imposing additional physical and mental workloads.

**Objectives:**

We aimed to evaluate the impact of the pandemic on the professional quality of life of HCWs.

**Methods:**

We conducted a cross-sectional study among HCWs working in the Covid-19 unit in Sfax, Tunisia in December 2021. We used a self-administrated questionnaire including sociodemographic and professional information. Professional issues were assessed by The Professional Quality of Life scale.

**Results:**

Our population consisted of 69 participants. The mean age was 31± 6 years. The sex ratio (M/F) was 1.1. Sixty-eight per cent had a university education. On a scale from 0 to 10,76.8% rated their Health Status greater than or equal to 8 and the mean score was 8.89±1. Medical history of Covid-19 infection was found in 37.7% of paricipants and 94,1% were vaccinated against SARS Cov 2.

Most of the workers showed moderate to high levels of compassion (65.2% and 31.9 % respectively). Fifty-five per cent showed moderate burnout levels. Only 2.9% of the population had a high level of secondary traumatic stress.

Secondary traumatic stress was associated with age (p=0.049; R=0.238). The males were more affected with burnout symptoms. Vaccination Status was associated with compassion satisfaction (p=0.042). Health Status Evaluation was not correlated with compassion satisfaction, burnout or secondary traumatic stress.

**Conclusions:**

The current pandemic has affected the HCWs system professional and social lives. A long follow-up should be maintained to support HCWs dealing with the pandemic.

**Disclosure of Interest:**

None Declared

